# Prevalence of diabetic retinopathy and diabetic macular edema in a primary care-based teleophthalmology program for American Indians and Alaskan Natives

**DOI:** 10.1371/journal.pone.0198551

**Published:** 2018-06-20

**Authors:** Sven-Erik Bursell, Stephanie J. Fonda, Drew G. Lewis, Mark B. Horton

**Affiliations:** 1 Telehealth Research Institute, John A. Burns School of Medicine, University of Hawaii, Honolulu, Hawaii, United States of America; 2 Estenda Solutions, Inc., Conshohocken, Pennsylvania, United States of America; 3 Phoenix Indian Medical Center, Phoenix, Arizona, United States of America; University of Florida, UNITED STATES

## Abstract

**Background:**

Diabetes and its complications are more common in American Indians and Alaska Natives (AI/AN) than other US racial/ethnic populations. Prior reports of diabetic retinopathy (DR) prevalence in AI/AN are dated, and research on diabetic macular edema (DME) is limited. This study characterizes the recent prevalence of DR and DME in AI/AN using primary care-based teleophthalmology surveillance.

**Methods:**

This is a multi-site, clinic-based, cross-sectional study of DR and DME. The sample is composed of AI /AN patients with diabetes (n = 53,998), served by the nationally distributed Indian Health Service-Joslin Vision Network Teleophthalmology Program (IHS-JVN) in primary care clinics of US Indian Health Service (IHS), Tribal, and Urban Indian health care facilities (I/T/U) from 1 November 2011 to 31 October 2016. Patients were recruited opportunistically for a retinal examination using the IHS-JVN during their regular diabetes care. The IHS-JVN used clinically validated, non-mydriatic, retinal imaging and retinopathy assessment protocols to identify the severity levels of non-proliferative diabetic retinopathy (NPDR), proliferative diabetic retinopathy (PDR), DME, and sight threatening retinopathy (STR; a composite measure). Key social-demographic (age, gender, IHS area), diabetes-related health (diabetes therapy, duration of diabetes, A1c), and imaging technology variables were examined. The analysis calculated frequencies and percentages of severity levels of disease.

**Results:**

Prevalence of any NPDR, PDR, DME, and STR among AI/AN patients undergoing DR teleophthalmology surveillance by IHS-JVN was 17.7%, 2.3%, 2.3%, and 4.2%, respectively. Prevalence was lowest in Alaska and highest among patients with A1c >/ = 8%, duration of diabetes > 10 years, or using insulin.

**Conclusions:**

Prevalence of DR in this cohort was approximately half that in previous reports for AI/AN, and prevalence of DME was less than that reported in non-AI/AN populations. A similar reduction in diabetes related end-stage renal disease in the same population and time period has been reported by other researchers. Since these two diabetic complications share a common microvasculopathic mechanism, this coincident change in prevalence may also share a common basis, possibly related to improved diabetes management.

## Introduction

American Indians and Alaska Natives (AI/AN) have an age-adjusted prevalence of diagnosed diabetes that is 2.0 times that of non-Hispanic whites. Prevalence varies by region from 6.0% among AN to 22.2% among AI in certain areas of the Southwest [1]. Diabetic retinopathy (DR) is the most common microvascular complication of diabetes [2]. The few published studies of DR in AI/AN populations have documented prevalence rates of 35% to 49% for non-proliferative diabetic retinopathy (NPDR) and 3% to 10% for proliferative diabetic retinopathy (PDR) [3–6]. These studies, conducted in the 1980s and 1990s individually included cohorts living in the US Southwest, Northern Plains, or Oklahoma. Similarly, in a Canadian First Nations Indian population, the prevalence of NPDR reported in 2007 was approximately 40% [7]. The prevalence of diabetic macular edema (DME) has not been reported among AI/AN.

DR [8] and DME [9] are leading causes of severe and moderate vision loss among working age adults in the US despite the effectiveness of timely diagnosis and treatment. Because approximately half the AI/AN population with diabetes fails to obtain the recommended annual retinal examination required to allow appropriate DR management, the Indian Health Service (IHS) implemented a primary care-based teleophthalmology program in 2000 to increase compliance with DR surveillance standard of care [10], and improve efficiencies [11].

This particular program, called the IHS-Joslin Vision Network Teleophthalmology Program (IHS-JVN) [12], uses a non-mydriatic retinal imaging protocol validated to American Telemedicine Association Category 3 [13–16]. The program has a diagnostic accuracy sufficient to determine levels of DR and DME commensurate with the Early Treatment Diabetic Retinopathy Study (ETDRS) clinical evaluation [15,16]. The IHS is a patient centered, public health-focused, federal health care organization serving AI/ANs in 35 states. AI/AN experience significant disparities in access to specialty care, in part due to their disproportionately rural distribution [17]. IHS-JVN has been integrated within existing IHS diabetes care programs to assess DR and DME without need for specialty referral or pupil dilation, thereby improving access to DR surveillance critical for timely management and prevention of avoidable vision loss [10].

The present study analyzed data from the IHS-JVN to characterize the prevalence of DR and DME among AI/AN undergoing teleophthalmology surveillance. Previous studies of DR prevalence in AI/AN populations were limited to only a few geographic areas, had limited tribal representation, and did not address DME. In addition, most were published two to three decades prior to the outcomes of clinical trials demonstrating reduction of diabetes related end-organ disease by improved diabetes management [18–21]. This study expands and updates the literature on these retinal conditions among AI/AN, with implications for health care providers serving AI/AN, regulators, and for Indigenous populations globally.

## Materials and methods

### Sample

This was a retrospective data analysis of AI/AN persons with diabetes evaluated by the IHS-JVN. The IHS-JVN was started in 2000 and took its first images as a distributed clinical program in 2002. As of September 2017, the IHS-JVN was operational in 11 of the 12 IHS administrative areas spanning the United States, deployed in 142 IHS, Tribal, and Urban clinics, and conducted over 162,122 evaluations for DR (also known as “studies”). The IHS-JVN program is estimated to have a catchment population of 60–70% of AI/AN with known DM [22]. Thus, the IHS-JVN is an extensive program with a nationally geographic pattern that includes broad tribal representation and aligns with the frequency distribution of AI/AN people in the United States. This acts to minimize the impact of geographic and tribal variations in DR that is present in all previous reports of DR prevalence among AI/AN, as well as the impact of locations not served by the IHS-JVN.

This retrospective data analysis examines the cohort of patients evaluated by IHS-JVN from 1 November 2011 to 31 October 2016. The total number of facilities participating in the IHS-JVN in this timeframe was 96. Typically, these facilities are the predominate or only source of diabetes care available to AI/AN patients in the community. They are staffed by community health nurses and support staff who facilitate patient attendance to services, particularly patients with chronic disease like diabetes. In each of these facilities, patients with diabetes were recruited for retinal imaging consecutively during their primary care appointments, as a routine component of their standard diabetes care. Thus, evaluation by IHS-JVN did not require formal referral or a separate appointment, thereby mitigating barriers to access and the associated selection bias.

### Ethics statement

This analysis was approved by the IHS National Investigational Review Board. The data were de-identified in accordance with Health Insurance Portability and Accountability Act (HIPAA) regulations.

### Technology and protocol

The IHS-JVN uses two technical configurations for acquisition of retinal images. Both have been validated against the gold standard of ETDRS 7 standard fields photography. The first configuration uses a 45° (degree) field of view (FOV), low-illumination, nonmydriatic fundus photography (NMFP) digital imaging system (Topcon NW6S; Topcon Medical Systems, Inc., Paramus, NJ) with a custom digital camera back that is effective in low-light conditions (Megavision Retinal Image Capture; Santa Barbara, CA) [16]. Using the NMFP system, three 45° and two 30° FOV stereo pair digital images of the retina and one external image of the anterior segment for each eye are obtained by certified imagers as previously described [15]. Silva and colleagues found this configuration has near perfect agreement with the gold standard of ETDRS photography for diagnosis of DR severity levels (κ = 0.81, 95% confidence interval = 0.73–0.89) [16]. The second configuration uses nonmydriatic ultrawide-field (UWFI) scanning laser ophthalmoscopy (SLO) (Daytona, Optos, plc, Dunfermline, United Kingdom). It was first introduced in the IHS-JVN 1 October 2014 [14], and staged incrementally across 21 sites as upgrades to previous NMFP systems. The UWFI protocol includes a single macula-centered 200° FOV stereo pair image from each eye. Silva and colleagues found exact DR severity agreement between UWFI and ETDRS photography occurred in 84% of cases, and agreement within one level of severity occurred in 91% of cases (weighted κ = 0.85 and unweighted κ = 0.79) [23]. More IHS clinics will adopt UWFI over time, but currently the IHS-JVN uses both technologies because NMFP is less costly and relatively portable, allowing the program to access smaller and more remotely located populations that would otherwise not be represented here.

The retinal images were securely transmitted to a central reading center for grading according to a standard validated protocol by certified optometrist tele-retinal readers with ophthalmologist supervision. The IHS-JVN reading software utilized computer assisted decision support based upon ETDRS criteria to further facilitate standardized grading of the retinal images. Pertinent health information for each patient was obtained from the IHS electronic health record (EHR). The reader renders a diagnosis of DR and DME severity and recommends a management plan in a report sent to the patient’s primary care provider (or designee) for further management of the patient. Ongoing quality assurance through a structured process of monthly administrative and clinical review provided an evidence basis for continued reader certification, and ongoing clinical performance consistent with the program’s original validation studies.

For the present study, diagnoses for both eyes were combined to create a person-level diagnosis representing the most severe level of DR and DME observed. If the diagnoses between a patient’s eyes differed, the diagnosis from the eye that indicated the most severe level of disease was selected. Each image underwent rule-based assessment for gradability [24]. If images for one eye could not be interpreted due to the technical quality of the images, but images for the fellow eye could be interpreted conclusively, the diagnosis from the interpreted images was used.

If a patient was imaged more than once in the five-year timeframe, the case indicating the most severe level of DR or DME was recorded. Otherwise, if the patient had no signs of DR or DME, or all records during the selected timeframe documented the same level of disease, the earliest case was recorded. Therefore, each patient is represented in the dataset only once.

### Measures

#### Outcomes

The IHS-JVN identified the severity levels of DR and DME based on ETDRS criteria and consistent with the International Classification System for Diabetic Retinopathy and Macular Edema [25].

If the technical quality of the images was insufficient to make a conclusive determination of the level of DR or DME, the imager could not obtain all images for the patient, or there were technical difficulties resulting in an incomplete imaging protocol, the reader reported them as ‘ungradable’ for that condition. Because stereo imaging and overlapping retinal fields provided redundancy of data within a single retinal field, images may be ungradable for one condition, but sufficient to obtain a grade for others. Except for an initial examination of background characteristics, patients with ungradable images are excluded from the analyses, consistent with previous research [26,27]. A case designation of ‘ungradable’ resulted in an automatic referral for a conventional dilated retinal exam.

For DR, the possible outcomes were: 1) no apparent DR; 2) mild NPDR, meaning microaneurysms only; 3) moderate NPDR, meaning more than microaneurysms but less than severe NPDR; 4) severe NPDR, indicated by intra-retinal hemorrhage in each of the four quadrants or venous beading in two or more quadrants or intraretinal microvascular abnormalities (IRMAs) in one or more quadrants, but no PDR; and 5) evidence of PDR, indicated by neovascularization and/or vitreous preretinal hemorrhage.

For DME, the outcomes were: 1) absent; 2) not clinically significant, characterized by retinal thickening or hard exudates at or within 3,000 microns from the fovea or thickening in the posterior pole within the arcades that is outside the threshold for Clinically Significant Diabetic Macular Edema (CSDME); and 3) CSDME, characterized by retinal thickening at or within 500 microns of the fovea, hard exudates at or within 500 microns of the fovea with adjacent retinal thickening, one or more disc areas of retinal thickening any part of which is within 1,500 microns of the fovea or with center involvement [28].

We also determined the outcome for sight threatening retinopathy (STR), defined as present if severe NPDR and/or PDR and/or any DME was evident.

#### Health summary data

The IHS-JVN readers were presented with a five-year health summary that is obtained automatically from the IHS EHR and supplemented as needed by the imager. The summary includes evidence-based risk factors for the progression of DR [9,29] to guide the care plan. The health summary data included glycemic control, body mass index (BMI), smoking status, family history of diabetes, and presence of hypertension, hypercholesterolemia, nephropathy, and peripheral neuropathy. However, for health summary data accessible to this study, glycemic control was the most reliably recorded datum. Glycemic control was characterized as: A1c of less than 6%; A1c of 6% to 7.9%; A1c of 8% to 10%; A1c of greater than 10%; actual A1c not recorded, but ‘poor glycemic control’ is recorded; or missing.

The IHS-JVN software provides automated collection of diabetes duration from the EHR based upon time of diagnosis, rather than the patients’ recollection. Duration of diabetes is presented as a categorical variable. The software also collects information on the patients’ diabetes treatment, which includes diet only, oral diabetes medications, insulin only, insulin and oral medications, or unknown.

#### Social-demographics and technology used

The IHS-JVN software provides automated collection of demographic information from the patient’s EHR, such as age, gender, and imaging clinic name presented as categorical variables. Clinics were matched to the IHS’s twelve administrative areas and then consolidated into the following geographical areas: Southwest, Oklahoma, Northwest, Northern Plains, East of the Mississippi River, and Alaska. The type of technology used (NMFP or UWFI) was also documented.

### Statistical analysis

First, the IHS-JVN cohort was characterized by calculating frequencies, column and row percentages (to show the conditional distributions) for the social-demographics, and health summary and technology variables. Although the focus of this analysis is patients with gradable images, consistent with previous research, the analyses compared the conditional distributions of whether images were gradable or ungradable using chi-square tests. Second, to obtain overall prevalence estimates, the analyses calculated the numbers and column percentages of the IHS-JVN population for each DR, DME, and STR severity level. Third, the analyses calculated the frequencies and percentages for each level of DR, DME, and STR by social-demographics, health summary data, and technology used, and conducted chi-square tests of independence. Lastly, although not the primary focus of this study, the analyses estimated multinomial logit models with all aforementioned variables (not shown, but available upon request) to document their net effects on DR and DME. All analyses were done using SAS 9.4 (Cary, NC).

## Results

In the examined timeframe, 53,998 patients were imaged, 86.3% of which had gradable images (Table 1). Of those with gradable images, 40.3% were under age 50 and 8.8% were 70 years and older (mean age = 52.7± 12.8 years). The majority of patients were female (56.0%) and lived in the Southwest (57.6%). About 31.7% of patients had a diagnosis of diabetes for more than 10 years. Most patients were on oral medications alone for their diabetes therapy (51.5%). The most recent A1c was less than 6.0% (42 mmol/mol) for 9.4% of patients, 6.0 to 7.9% (42 to 63 mmol/mol) for 35.4% of patients, and 8% (64 mmol/mol) or greater for 38.2% of patients. For the remainder of patients, ‘poor glycemic control’ was noted or A1c data were missing.

**Table 1:** Characteristics of the IHS-JVN patients, by gradable or ungradable images.

Characteristic	Patients with gradable images for DR or DME			Patients with ungradable images for both DR and DME		Hypothesis tests of independence, gradable vs. ungradable		
	(n = 46584)			(n = 7414)		(n = 53998)		
	n	Column %	Row %	n	Row %	Chi-Sq	(df)	P-value
**Social-demographics**								
Age								
Less than 50 years	18793	40.3	93.2	1381	6.8	2844.7	(3)	< 0.0001
50 to 59 years	13977	30.0	88.6	1805	11.4			
60 to 69 years	9737	20.9	80.7	2324	19.3			
70 years and older	4077	8.8	68.2	1904	31.8			
Gender*								
Male	20476	44.0	85.0	3627	15.0	64.3	(2)	< 0.0001
Female	26105	56.0	87.3	3786	12.7			
Area								
Southwest	26829	57.6	87.0	4008	13.0	191.2	(5)	< 0.0001
Alaska	499	1.1	88.3	66	11.7			
East of Mississippi River	1743	3.7	79.0	462	21.0			
Northern Plains	4401	9.4	82.4	941	17.6			
Northwest	5326	11.4	86.8	807	13.2			
Oklahoma	7786	16.7	87.3	1130	12.7			
**Health summary data**								
Duration of diabetes								
Less than 1 year	5780	12.4	91.9	511	8.1	1015.2	(4)	< 0.0001
1 to 5 years	12862	27.6	90.5	1347	9.5			
6 to 10 years	9237	19.8	88.3	1220	11.7			
More than 10 years	14769	31.7	80.1	3668	19.9			
Unknown/missing	3936	8.4	85.5	668	14.5			
Diabetes therapy								
Diet only	4287	9.2	87.8	593	12.2	373.3	(4)	< 0.0001
Oral medications	23990	51.5	88.4	3152	11.6			
Insulin only	5467	11.7	80.5	1324	19.5			
Insulin & oral medications	8638	18.5	83.5	1710	16.5			
Unknown/missing	4202	9.0	86.9	635	13.1			
Glycemic control								
A1c of less than 6% (< 42 mmol/mol)	4393	9.4	87.3	638	12.7	77.9	(5)	< 0.0001
A1c of 6 to 7.9% (42 to 63 mmol/mol)	16499	35.4	86.9	2488	13.1			
A1c of 8 to 10% (64 to 86 mmol/mol)	8788	18.9	86.2	1409	13.8			
A1c of greater than 10% (> 86 mmol/mol)	8980	19.3	87.2	1320	12.8			
Missing but poor glycemic control noted	797	1.7	82.6	168	17.4			
Unknown/missing	7127	15.3	83.7	1391	16.3			
**Technology**								
NMFP	30049	64.5	81.2	6948	18.8	2529.8	(1)	< 0.0001
UWFI**	16535	35.5	97.3	466	2.7			

Column percentages are shown to characterize the analytic sample, patients with gradable images only.

Row percentages are shown to examine differences in the likelihood of having gradable images, based on patient characteristics.

All chi-square tests examining likelihood of having gradable versus ungradable images (by characteristic) indicate the 2 groups differ along these characteristics.

*3 people were 'other' for gender and were excluded.

**UWFI became available in this program Sept 2014.

IHS-JVN = Indian Health Service-Joslin Vision Network Teleophthalmology Program; DR = diabetic retinopathy; DME = diabetic Macular Edema; NMFP = nonmydriatic flash photography; UWFI = ultrawide-field imaging; df = degrees of freedom.

Patients with gradable images tended to be younger, female, had shorter duration of diabetes, not taking insulin, and had a recorded value for A1c (i.e., not missing data). Because the clinics east of the Mississippi River were more recent additions to the IHS-JVN and were smaller, the imagers were less experienced and the area’s percentage of gradable images was lower. All chi-square tests comparing the characteristics of patients with gradable images versus the characteristics of patients with ungradable images were statistically significant, rejecting the null hypothesis of no differences between groups.

The total percentage of patients with any DR was 20.0% (Table 2), with 17.7% having NPDR and 2.3% having PDR. Prevalence of any DME was 2.3%. Prevalence of STR was 4.2%.

**Table 2:** Numbers (n) and percentages (%) of IHS-JVN patients by level of DR and DME.

Severity level	n	%
**DR**		
No Apparent DR	36381	80.0
Mild NPDR	4284	9.4
Moderate NPDR	3698	8.1
Severe NPDR	67	0.1
PDR	1052	2.3
*Total*	45482	100.0
**DME**		
Absent	44806	97.7
Not Clinically Significant	653	1.4
CSDME	394	0.9
*Total*	45853	100.0
**STR**		
Absent	43055	95.8
Present	1904	4.2
*Total*	44959	100.0

Excludes patients with ungradable images.

The different total n for DR and DME is due to differing ungradable rates.

IHS-JVN = Indian Health Service-Joslin Vision Network; Teleophthalmology Program; DR = diabetic retinopathy; NPDR = nonproliferative diabetic retinopathy; PDR = proliferative diabetic retinopathy; DME = diabetic macular edema; CSDME = clinically significant DME; STR = sight threatening retinopathy.

Compared with other age groups, a higher percentage of patients aged 60 years and older had mild NPDR (Table 3), and a higher percentage of patients less than age 60 years had moderate NPDR. A slightly higher percentage of males had moderate NPDR. With respect to geography, the highest percentage of patients with no DR was in Alaska, whereas the highest percentage of patients with PDR was in the Southwest. The percentages of people with any level of DR greater than ‘no apparent’ increased in expected ways when risk factors were considered; i.e., percentages were higher among patients with longer duration of diabetes and patients taking insulin alone or with oral medications. Higher percentages of mild and moderate NPDR were found using UWFI than NMFP, but there was no difference in percentage of severe NPDR. UWFI identified PDR twice as frequently as did NMFP.

**Table 3:** Numbers (n) and percentages (%)* of DR severity level among IHS-JVN patients, by their characteristics.

Characteristic	No Apparent DR		Mild NPDR		Moderate NPDR		Severe NPDR		Evidence of PDR		Hypothesis tests of independence		
	n	%	n	%	n	%	n	%	n	%	Chi-Sq	(df)	P-value
Age													
Less than 50 years	14826	80.5	1440	7.8	1692	9.2	38	0.2	413	2.2	292.2	(12)	< 0.0001
50 to 59 years	10868	79.6	1260	9.2	1195	8.7	20	0.1	315	2.3			
60 to 69 years	7576	79.9	1031	10.9	630	6.6	6	0.1	233	2.5			
70 years and older	3111	79.0	553	14.0	181	4.6	3	0.1	91	2.3			
Gender**													
Male	15525	77.9	1921	9.6	1953	9.8	27	0.1	495	2.5	147.4	(4)	< 0.0001
Female	20853	81.6	2363	9.2	1745	6.8	40	0.2	557	2.2			
Area													
Southwest	20508	78.0	2516	9.6	2559	9.7	35	0.1	674	2.6	317.4	(20)	< 0.0001
Alaska	452	92.2	25	5.1	10	2.0	0	0.0	3	0.6			
East of Mississippi River	1357	82.3	154	9.3	100	6.1	1	0.1	37	2.3			
Northern Plains	3455	80.6	399	9.3	324	7.6	9	0.2	99	2.3			
Northwest	4279	82.9	458	8.9	337	6.5	8	0.2	78	1.5			
Oklahoma	6330	83.2	732	9.6	368	4.8	14	0.2	161	2.1			
Duration of diabetes													
Less than 1 year	5362	94.5	148	2.6	133	2.3	3	0.1	31	0.5	5901.4	(16)	< 0.0001
1 to 5 years	11713	92.9	475	3.8	367	2.9	4	0.0	52	0.4			
6 to 10 years	7508	83.2	786	8.7	610	6.8	13	0.1	103	1.1			
More than 10 years	8569	59.8	2593	18.1	2351	16.4	43	0.3	777	5.4			
Unknown/missing	3229	84.1	282	7.3	237	6.2	4	0.1	89	2.3			
Diabetes therapy													
Diet only	3971	94.5	124	3.0	72	1.7	0	0.0	34	0.8	4588.9	(16)	< 0.0001
Oral medications	20393	87.0	1543	6.6	1263	5.4	17	0.1	232	1.0			
Insulin only	2994	56.3	1017	19.1	906	17.0	20	0.4	382	7.2			
Insulin & oral medications	5466	65.0	1358	16.2	1230	14.6	25	0.3	328	3.9			
Unknown/missing	3557	86.6	242	5.9	227	5.5	5	0.1	76	1.9			
Glycemic control													
A1c of less than 6% (< 42 mmol/mol)	3974	92.7	155	3.6	101	2.4	0	0.0	55	1.3	2416.0	(20)	< 0.0001
A1c of 6 to 7.9% (42 to 63 mmol/mol)	14077	87.2	1180	7.3	619	3.8	9	0.1	265	1.6			
A1c of 8 to 10% (64 to 86 mmol/mol)	6195	72.4	1191	13.9	883	10.3	18	0.2	269	3.1			
A1c of greater than 10% (> 86 mmol/mol)	5934	67.5	1108	12.6	1428	16.2	29	0.3	293	3.3			
Missing but poor glycemic control noted	597	77.4	87	11.3	72	9.3	3	0.4	12	1.6			
Unknown/missing	5604	80.9	563	8.1	595	8.6	8	0.1	158	2.3			
Technology													
NMFP	24557	84.6	2345	8.1	1590	5.5	50	0.2	480	1.7	1214.8	(4)	< 0.0001
UWFI	11824	71.8	1939	11.8	2108	12.8	17	0.1	572	3.5			

Excludes patients with ungradable images; All chi-square tests examine the likelihood of having a certain severity level of DR.

* Row percentage;

**3 people were 'other' for gender and were excluded.

IHS-JVN = Indian Health Service-Joslin Vision Network Teleophthalmology Program; DR = diabetic retinopathy; NPDR = nonproliferative diabetic retinopathy;

NMFP = nonmydriatic flash photography; UWFI = ultrawide-field imaging; df = degrees of freedom.

The percentages of patients with DME (not clinically significant) were higher in patients less than 60 years, male, had diabetes for more than 10 years, taking insulin, and with higher A1c or poor glycemic control (Table 4). The percentages of patients with CSDME were higher in patients who had diabetes for more than 10 years, were taking insulin only, and had an A1c of greater than 10%. Rates of DME (not clinically significant) and CSDME were lowest in Alaska; otherwise, there was no meaningful difference by area. There was no difference between NMFP and UWFI in the percentage of patients found to have any DME.

**Table 4:** Numbers (n) and percentages (%)*of level of DME among IHS-JVN patients, by their characteristics.

Characteristic	Absent		Not Clinically Significant		CSDME		Hypothesis tests of independence		
	n	%	n	%	n	%	Chi-Sq	(df)	P-value
Age									
Less than 50 years	18217	97.7	284	1.5	139	0.7	48.6	(6)	< 0.0001
50 to 59 years	13410	97.2	242	1.8	141	1.0			
60 to 69 years	9343	98.1	106	1.1	76	0.8			
70 years and older	3836	98.5	21	0.5	38	1.0			
Gender**									
Male	19585	97.4	336	1.7	187	0.9	17.8	(2)	0.0001
Female	25218	98.0	317	1.2	207	0.8			
Area									
Southwest	25787	97.6	402	1.5	221	0.8	14.3	(10)	0.1617
Alaska	492	99.4	2	0.4	1	0.2			
East of Mississippi River	1685	98.1	18	1.0	15	0.9			
Northern Plains	4209	97.7	66	1.5	35	0.8			
Northwest	5123	97.6	72	1.4	52	1.0			
Oklahoma	7510	97.9	93	1.2	70	0.9			
Duration of diabetes									
Less than 1 year	5696	99.1	29	0.5	21	0.4	556.5	(8)	< 0.0001
1 to 5 years	12669	99.2	63	0.5	36	0.3			
6 to 10 years	8965	98.3	101	1.1	53	0.6			
More than 10 years	13703	95.4	414	2.9	253	1.8			
Unknown/missing	3773	98.0	46	1.2	31	0.8			
Diabetes therapy									
Diet only	4225	99.4	14	0.3	11	0.3	504.3	(8)	< 0.0001
Oral medications	23388	98.6	205	0.9	134	0.6			
Insulin only	4995	94.7	173	3.3	107	2.0			
Insulin & oral medications	8108	95.9	231	2.7	120	1.4			
Unknown/missing	4090	98.7	30	0.7	22	0.5			
Glycemic control									
A1c of less than 6% (< 42 mmol/mol)	4296	99.3	16	0.4	14	0.3	420.9	(10)	< 0.0001
A1c of 6 to 7.9% (42 to 63 mmol/mol)	16086	98.9	89	0.5	85	0.5			
A1c of 8 to 10% (64 to 86 mmol/mol)	8376	97.2	156	1.8	86	1.0			
A1c of greater than 10% (> 86 mmol/mol)	8415	95.3	279	3.2	134	1.5			
Missing but poor glycemic control noted	760	96.0	21	2.7	11	1.4			
Unknown/missing	6873	97.8	92	1.3	64	0.9			
Technology									
NMFP	29014	97.8	416	1.4	241	0.8	2.49	(2)	0.2876
UWFI	15792	97.6	237	1.5	153	0.9			

Excludes patients with ungradable images; All chi-square tests examine the likelihood of having a certain severity level of DME.

* Row percentage;

**3 people were 'other' for gender and were excluded.

IHS-JVN = Indian Health Service-Joslin Vision Network Teleophthalmology Program; DME = diabetic macular edema;

CSDME = clinically significant diabetic macular edema; NMFP = nonmydriatic flash photography; UWFI = ultrawide-field imaging; df = degrees of freedom.

STR was highest for patients less than 70 years of age, males, patients with longer duration of diabetes, patients taking insulin, patients with higher A1c or missing A1c data in the IHS-JVN record, and patients imaged with UWFI (Table 5). It was lowest in people who live in Alaska.

**Table 5:** Numbers (n) and percentages (%)* of STR among IHS-JVN patients, by their characteristics.

Characteristic	No STR Apparent		STR Apparent		Hypothesis tests of independence		
	n	%	n	%	Chi-Sq	(df)	P-value
Age							
Less than 50 years	17585	95.9	759	4.1	8.7	(3)	0.0337
50 to 59 years	12914	95.4	627	4.6			
60 to 69 years	8928	95.9	377	4.1			
70 years and older	3628	96.3	141	3.7			
Gender**							
Male	18754	95.4	913	4.6	14.3	(1)	0.0002
Female	24298	96.1	991	3.9			
Area							
Southwest	24816	95.5	1174	4.5	23.6	(5)	0.0003
Alaska	481	98.8	6	1.2			
East of Mississippi River	1561	96.0	65	4.0			
Northern Plains	4041	95.6	184	4.4			
Northwest	4912	96.4	186	3.6			
Oklahoma	7244	96.2	289	3.8			
Duration of diabetes							
Less than 1 year	5580	98.7	72	1.3	556.5	(8)	< 0.0001
1 to 5 years	12388	98.9	137	1.1			
6 to 10 years	8688	97.3	243	2.7			
More than 10 years	12775	90.7	1305	9.3			
Unknown/missing	3624	96.1	147	3.9			
Diabetes therapy							
Diet only	4114	98.7	53	1.3	1358.1	(4)	< 0.0001
Oral medications	22712	97.8	515	2.2			
Insulin only	4610	88.5	599	11.5			
Insulin & oral medications	7677	92.5	621	7.5			
Unknown/missing	3942	97.1	116	2.9			
Glycemic control							
A1c of less than 6% (< 42 mmol/mol)	4149	98.2	78	1.8	439.4	(5)	< 0.0001
A1c of 6 to 7.9% (42 to 63 mmol/mol)	15569	97.6	389	2.4			
A1c of 8 to 10% (64 to 86 mmol/mol)	7972	94.5	467	5.5			
A1c of greater than 10% (> 86 mmol/mol)	8060	92.6	643	7.4			
Missing but poor glycemic control noted	727	94.3	44	5.7			
Unknown/missing	6578	95.9	283	4.1			
Technology							
NMFP	27782	96.4	1040	3.6	77.7	(1)	< 0.0001
UWFI	15273	94.6	864	5.4			

Excludes patients with ungradable images; All chi-square tests examine the likelihood of having STR.

* Row percentage;

**3 people were 'other' for gender and were excluded.

IHS-JVN = Indian Health Service-Joslin Vision Network Teleophthalmology Program; STR = sight threatening retinopathy; NMFP = nonmydriatic flash photography; UWFI = ultrawide-field imaging; df = degrees of freedom.

Multinomial logistic regression analyses documented that the net, statistical associations of all the variables included were as expected given prior research on the progression of DR and DME [9,29].

## Discussion

This study provides data on prevalence and severity of DR and DME in AI/AN who had undergone primary care based retinal imaging by the IHS-JVN from 1 November 2011 to 31 October 2016. The IHS-JVN program is validated, standardized, has robust quality assurance to ensure ongoing fidelity with validation studies, and provides recent retinal imaging data and pertinent medical record information for a geographically representative population of AI/AN, which predominately has type 2 diabetes. The expansive geographic scope of the IHS-JVN, federal funding of IHS health care, and opportunistic recruitment of patients from diabetes primary care workflow mitigates bias in DR prevalence due to local factors such as diet, difference in socio-economic status, health care utilization, and patient selection.

There are few studies of DR in AI/AN populations. Many AI/AN populations live in remote areas, making clinical studies of broad geographical scope difficult. Most of the previous studies are over two decades old. The results from these prior studies are summarized in Table 6.

**Table 6:** Reports of the prevalence of DR and/or DME in studies of native populations.

**Lead Author [Reference Number]**		Rate [4]	Newell [6]	Nagi [3]	Berinstein [5]	Lee [31]	Ross [7]
**Study Type**		Cross-sectional and clinic-based	Cross-sectional and clinic-based	Cross-sectional population based	Cross-sectional	Cross-sectional	Cross-sectional
**Population/Sample**	*Sample Size*	134	142	1030	417	1019	232
	*Tribe(s)*	Hopi (77) and Navajo (60)	Predominantly Cheyenne—Arapaho	Pima	Cheyenne River Sioux Tribe and the Oglala Sioux Tribe	Seven Oklahoma tribes	First Nations people of Canada
	*Location*	North-eastern Arizona, US	Clinton, Oklahoma, US	Gila River Indian Community, South central Arizona, US	Northern Plains, US	Oklahoma, US	Southern Alberta, Canada
	*Comments*	Patients at one IHS clinic	Patients at three IHS clinics	Included 288 people without diabetes	Diabetic participants in the Strong Heart Study	2^nd^ examination of the Strong Heart Study	
**Study Duration**		Jul 1979—Jun 1980	Oct 1985 –July 1987	Apr 1982-Dec 1990	1991	Sep 1995 –Mar 1998	1986
**Background Characteristics**	*Age (years)*	Range: >/ = 20	Average: 55.8; Range: 27 to 81	Average: 47; Range: 15 to 88	Range: 45–75. Average not reported.	Range: 48 to 82. 42.5%; aged 48–59; 36.5% aged 60–69; 21.0% aged 70–82	Not reported
	*% Male*	48.2	39.4	37	Not reported	39.8	Not reported
	*Diabetes Duration*	Average: 7.5 years (SD not reported)	0–5 years duration = 30.3%; 6–10 years = 26.1%; 11+ years = 33.8%; the rest unknown	9.1 years	Averages: 6.4 ± 7.9 years if no DR; 12.3 ± 7.5 if NPDR; 14.2 ± 10.3 if PDR	Not reported	Average: 8.26 ± 7.13 years
	*Medication*	40% taking oral medications; 37% taking insulin	50.7% taking oral medications only; 35.2% taking insulin with or without oral medications	28% taking oral medications; 34% taking insulin	Not reported	Not reported	Not reported
	*A1C (%)*	Not reported	Averages: 11.8 ± 3.8 if taking insulin; 12.4 ± 2.8 if not taking insulin	9.9 ± 2.6 years	Averages: 7.7 ± 2.6 if no DR; 8.9 ± 2.0 if NPDR; 9.3 ± 2.5 if PDR	Not reported	Average: 7.52 ± 2.0
**Prevalence**	*DR*	Any DR = 36%	Any DR = 49.3%. PDR = 21.4%	Any DR = 37.8%, PDR = 2.7%	NPDR = 40.2%; PDR = 5.1%	Any DR = 20.1%	Any DR = 39.7% PDR = 5.5%
	*DME*	Not reported	Not reported	Not reported	Not reported	DME not reported. CSDME = 2.6% (MD exam) or 2.4% (Reading Center exam)	Not reported
**Other Comments**		No difference in prevalence between the 2 tribes.		Data for people with diabetes only reported here.	Risk factors reported by presence or level severity of retinopathy.	DR prevalence not the primary outcome. Number of subjects with diabetes and factors pertinent to diabetes/DR not reported.	2247 was total sample size, with 232 being natives. Data for natives only reported here.

DR = diabetic retinopathy; NPDR = nonproliferative diabetic retinopathy; PDR = proliferative diabetic retinopathy; DME = diabetic macular edema; CSDME = clinically significant diabetic macular edema; Sight threatening retinopathy (STR) was reported in studies on nonnative populations, but not for the studies of native populations.

The prevalence of DR in these earlier studies was higher than that reported here, i.e., in Pima Indians with type 2 diabetes [3], Navajo and Hopi Indians [4], and Sioux Indian tribes [5], the prevalence of NPDR was 37.8%, 40%, and 45.3% respectively. The prevalence of PDR was 2.7% (Pima), and prevalence of vision-threatening retinopathy was 8.2% (Navajo and Hopi). A longitudinal (12.7 years) study in AI examined in Oklahoma found that the overall incidence of PDR among survivors was 18.6%, and 45% of those with background DR (NPDR) at baseline later developed PDR [30]. A 2005 study in Oklahoma showed a DR prevalence of 20.1% [31], which is more consistent with our 2011 to 2016 results (16.7%, Table 3) from that region.

These earlier AI/AN NPDR prevalence rates (ranging between 20% and 49%) are similar to non-native groups documented in the Wisconsin Epidemiologic Study of Diabetic Retinopathy (WESDR), where DR prevalence varied from 29% (duration of diabetes less than 5 years) to 78% (duration of diabetes greater than 15 years), and PDR varied from 2% (duration less than 5 years) to 15.5% (duration greater than 15 years) [32].

Analyses of DR prevalence in diabetic populations, both globally [29] and in the US [26,33], provide a benchmark, though none provide data on AI/AN populations. A meta-analysis on global prevalence of any DR (NPDR and/or PDR and/or DME) [29]—including a total of 35 studies (1980–2008) and data from 22,896 non-AI/AN individuals with diabetes (both type 1 and 2)—showed an overall prevalence of 34.6% for any DR, 7.0% for PDR, 6.8% for DME and 10.2% for STR (PDR and/or DME). For only individuals with type 2 diabetes, the prevalence for any DR was 25.2%, for PDR was 3.0%, and for STR was 6.9%. In the US, an analysis of 2005–2008 National Health and Nutrition Examination Survey (NHANES) data [26] showed a prevalence of DR (NPDR plus PDR) of 28.5%, PDR of 1.5%, CSDME of 2.7% and STR (Severe NPDR, PDR, and CSDME) of 4.4% in a non-AI/AN diabetic patient population over the age of 40.

The design of this study prevents identification of factors contributing to the lower prevalence of DR as compared with prior studies of AI/AN. However, evidence from studies of non-AI/AN populations suggest that improved diabetes management has resulted in a general decline in diabetes related end-organ disease [34]. In this context, it is noteworthy that the reduction in DR prevalence observed in this study is temporally associated with the IHS Special Diabetes Program for Indians (SDPI), initiated in 1997 [35,36]. The specific character of SDPI programs varied among health care facilities to suit local needs, but was otherwise uniformly available across all facilities serving AI/AN for the purpose of improving diabetes management, and in many instances included the IHS-JVN [36]. This systematic program of public health and population management approaches for diabetes care doubled, or in some cases tripled access to organizational diabetes health services, including diabetes clinics, diabetes clinical teams, nutrition services, culturally tailored diabetes education, physical activity specialists, and weight management programs [36]. It was associated with clinically significant improvements in A1c levels, blood lipid levels, and blood pressure [37], which are risk factors for onset and progression of DR and DME. For example, average A1c among AI/AN dropped from 9.0% in 1996 to 8.1% in 2010 [3]. These and other improvements in diabetes management coincident with the implementation of SDPI have been related to a 54% reduction in diabetes related end stage renal disease (dESRD) among AI/AN [37,38]. This is particularly relevant since dESRD and DR share a similar microvasculopathy etiology. Thus, it is not surprising to see a parallel improvement in prevalence of diabetes-related retinal disease in this population. Similar significant improvements in DR and other diabetes-related complications have recently been observed for non-AI/AN populations in the US and Europe [34,39].

To our knowledge, no other studies have investigated the prevalence of DME in the AI/AN population. Comparisons between this study and previous non-AI/AN studies are difficult because the criteria for DME and CSDME differ across the studies or are not defined. In the US, two separate studies using 2005 to 2008 NHANES data showed a DME prevalence rate of 3.8% in patients aged over 40 years [9] and a CSDME prevalence of 2.7% [24]. Further, in the Wisconsin epidemiologic study of diabetic retinopathy. IV [40], which included patients who were over 30 years of age at diagnosis, the prevalence rates of DME ranged from 3.0% (duration of diabetes less than 5 years) to 28.0% (duration greater than 20 years). In contrast, the Multi-Ethnic Study of Atherosclerosis (MESA) reported a 9.0% DME prevalence [27]. The AI/AN prevalence of DME reported here is lower than that reported in the published literature for non-AI/AN populations. In general, these studies depended upon monoscopic, surrogate indicators of macular edema rather than directly observed retinal thickening by stereoscopic viewing as used by the IHS-JVN. This has the effect of under-reporting DME determined by surrogate indicators in the absence of micro aneurysms or hard exudates, and under-reporting CSDME vs DME. However, studies have shown that this technique is reliable for identification of any level of DME [41,42]. Although a basis for the lower rate of DME observed in this study cannot be established, this difference, in part, may be due to the aforementioned improvements in diabetes management, a potential protective effect of the retinal pigmentation in AN/AI [43], and/or other racial/ethnic differences in the nature of DME.

A potential limitation of this study is selection bias. First, patients with more advanced retinal conditions may already be under specialty ophthalmological care and may have chosen to defer IHS-JVN as a result. Also, patients with diabetic retinal disease may also have poor glycemic control (A1c >7%) and would be at higher risk of mortality [44]. Both possibilities could result in some underreporting of DR and/or DME prevalence. Second, the sample may be biased in that the IHS-JVN accesses people who go to primary care clinics in facilities that participated in the teleophthalmology program. However, due to the aforementioned reach and geographic distribution of the program, and the fact that its services are offered to all known patients with diabetes receiving care at the facility, we believe risk of this type of bias is low. Indeed, unlike many previous reports of DR and DME prevalence, these patients were not recruited in specialty eye clinics, so this powerful source of selection bias was mitigated.

Another potential limitation of this study is that images for some patients were ungradable. Except for glycemic control, factors associated with the outcome of ‘ungradable’ paralleled those for risk of DR and DME [42, 45]. Thus, presence of a risk factor is more likely to result in ungradable images and referrals. But a designation of ungradable does not necessarily mean disease is visible or clinically overt. Images may be ungradable because of smaller pupil size and media opacity, both of which are more common with increasing age and duration of diabetes [45]. To address the question of DR and DME prevalence among patients who had ungradable images in the present analysis, we extracted the diagnoses codes from the medical records for a sample of 799 unique patients whose images were ungradable for DR and/or DME in 2013 and 2014 and who got a dilated eye exam within 365 days of that original ungradable finding. Of these patients 1.5% had NPDR “not otherwise specified” (i.e., level unknown), 2.4% had mild NPDR, 2.5% had moderate NPDR, 0.6% had severe NPDR, 2.6% had PDR, and 2.9% had DME. Another 24.0% had a diagnosis code indicating ‘background diabetic retinopathy’ only, with no NPDR severity level specified. These percentages indicate that the rate of sight-threatening diabetic eye disease is not substantially higher among patients who had ungradable images during the period of this study, whereas levels of DR that do not (yet) threaten sight might be. Further analysis of the outcomes for people who had ungradable images is a substantial, separate undertaking to be addressed in a future project.

To further address the question of DR and DME prevalence among patients with ungradable images, several of the tables herein reported findings from the NMFP and the UWFI separately. The ungradable rate using the NMFP was 18.8%, compared with 2.7% using UWFI, both of which are consistent with other reported studies [46]. Despite differences in ungradable rates, the NMFP and UWFI technologies yielded similar findings for prevalence and rates for severe NPDR and any DME in the present analysis, likely due to the more central occurrence of the condition, but the rate for PDR was twice as high with UWFI than with NMFP, likely resulting from the larger aggregate FOV that UWFI provides [46]. Other reports of UWFI imaging for DR suggest that it may be a more sensitive measure of DR in both native and non-native populations. [24,46] This is being explored by the Diabetic Retinopathy Clinical Research Network (DRCRnet) protocol AA [47], and its results may impact future standards for DR identification and risk stratification. Until then, the current standard for DR diagnosis remains ETDRS 7 standard fields photography, with which both technologies used by the IHS-JVN have high levels of agreement. Since all previous reports of DR prevalence were based upon central fields, direct comparisons of prevalence using UWFI is somewhat difficult, so comparison with earlier reports is best aligned technically with our NMFP data. Since our data includes more sensitive UWFI, this report may slightly overstate the prevalence as compared to legacy central field methods. This report shows a substantial decrease in the prevalence of DR using either UWFI or NMFP as compared to previous reports using central fields.

## Conclusions

To our awareness, this is the only systemic report of DR and DME among AI/AN, and updates older reports, which were limited to specific regions and AI/AN tribes. The AI/AN prevalence of NPDR, PDR, DME, and STR among AI/AN patients undergoing DR teleophthalmology surveillance by the IHS-JVN was 17.7%, 2.3%, 2.3%, and 4.2% respectively. This represents a decrease in DR prevalence of at least 50% compared to previous reports in the 1980s and 1990s. Identifying trends in DME prevalence is more problematic since comparators are not available for AI/AN, and reporting methods for DME is less standardized than DR. Nonetheless, the DME prevalence shown herein is lower than that in recent reports of non-AI/AN population. This study provides a benchmark for future research on DME in AI/AN.

The reduction in DR prevalence is temporally coincident with the implementation of the IHS SDPI, which has been similarly coincident with improvements in diabetic outcome metrics and dESRD. A simultaneous reduction in risk for these two major diabetes complications in this at risk population following the implementation of systemic programmatic changes in diabetes care provides clinicians, regulators, and federal funders possible further evidence for adherence to best practices of diabetes care. This information may be particularly helpful for clinicians, chronic disease program managers, and public health decision makers in the IHS and Tribal health care since it better defines a major chronic disease in AI/AN and facilitates the development of effective programs and provides a reliable baseline for evaluating the effects of programs over time.

Further research is required to clarify the impact of ungradable images on the prevalence of DR determined by the IHS-JVN, and to investigate links between improved diabetes care in the IHS population and reduced rates of retinal complications using IHS-JVN data available since 2000.

## Supporting information

S1 Table (XLSX)
